# Clinical Analysis of Neonatal Influenza in the Neonatal Intensive Care Unit: A Retrospective Study

**DOI:** 10.1002/iid3.70379

**Published:** 2026-02-17

**Authors:** Min Zhou, Jia Chen, Jing Zhao, Zhuli Yu, Mengjuan Feng, Xiang Qiu

**Affiliations:** ^1^ Department of Pediatrics Chengdu Integrated TCM & Western Medicine Hospital Chengdu Sichuan China; ^2^ Department of Ultrasound Chengdu Integrated TCM & Western Medicine Hospital Chengdu Sichuan China; ^3^ Department of Radiology Chengdu Integrated TCM & Western Medicine Hospital Chengdu Sichuan China

**Keywords:** influenza, maternal, neonate, oseltamivir, vaccination, virus

## Abstract

**Background:**

To describe the clinical characteristics, treatment, and outcomes of neonatal influenza.

**Methods:**

Retrospectively analyzed clinical data on 26 neonates who were diagnosed with neonatal influenza by positive influenza nasopharyngeal swab antigen tests, and admitted to the neonatal intensive care unit of Sichuan Province Chengdu Integrated TCM & Western Medicine Hospital in China, between January 2022 to December 2023.

**Results:**

Twenty‐six neonates were admitted, 25 with influenza A, whereas 1 co‐infected influenza A and B. Nine patients had close contact with family members showing respiratory symptoms prior to hospitalization. Common symptoms included fever (53.85%), nasal congestion (46.15%), cough, neonatal jaundice, and loss of appetite. The most frequent laboratory abnormalities were elevated CK‐MB (92.3%), lactic acid (80.77%), prominent lymphocytosis in both count and percentage, elevated monocyte percentage and AST. All patients treated with oseltamivir and recovered.

**Conclusion:**

In this single‐year study, influenza A was the predominant type identified. The clinical symptoms are non‐specific, and main symptom is fever 14/26 (53.85%). Treatment with oseltamivir is safe and had favorable outcomes.

## 1 Background

Influenza is an acute viral respiratory illness caused by infection of the respiratory tract with influenza viruses. As members of the Orthomyxoviridae family, influenza viruses are enveloped, negative‐sense single‐stranded RNA viruses. Influenza A and B viruses are responsible for seasonal epidemics and are commonly referred to as seasonal influenza viruses [1, 2, 3, 4, 5]. Infants and young children are highly susceptible to influenza virus infection [1, 2]. Especially during influenza epidemics, neonates are vulnerable to infection due to their immature immune systems and the absence of influenza vaccination, which increases their likelihood of developing severe illness. The clinical manifestations of neonatal influenza are often nonspecific and can even be asymptomatic in some cases, has an insidious onset, prolonged virus release, and high infectivity [1, 2, 3, 5, 6]. Nosocomial infection outbreaks can readily arise in the neonatal intensive care unit (NICU) if infected neonates are not promptly identified and isolated, potentially leading to grave outcomes [6]. Investigating the clinical characteristics, treatment approaches, and prevention of neonatal influenza is essential. Because of the small number of reported cases, current research remains limited. Hence, a retrospective analysis of clinical data was conducted on 26 newborns infected with the influenza virus in our hospital. This study aims to contribute further clinical evidence and to improve the diagnosis, treatment, and prevention of neonatal influenza.

## 2 Methods

The subjects of this study were neonates diagnosed with influenza by positive influenza nasopharyngeal swab antigen tests, and admitted to the neonatal intensive care unit of Sichuan Province Chengdu Integrated TCM & Western Medicine Hospital in China, from January 2022 to December 2023. All neonates were delivered in the obstetrics department of our hospital. Each neonate was initially discharged home before later required hospitalization for influenza. The following inclusion criteria were applied to patients: age ≤ 28 days, and diagnosis in accordance with the 2024 version of the “Expert Consensus on the Diagnosis and Treatment of Influenza in Children” influenza diagnostic criteria. The criteria of confirm influenza: a positive influenza virus nucleic acid test, a positive influenza antigen test, a positive influenza virus culture, and levels of influenza virus‐specific IgG antibodies in both acute and convalescent serum 4 or more times greater than normal [1, 7]. The exclusion criteria included serious underlying diseases, specifically congenital heart disease, genetic metabolic disease, or blood disease. Additionally, patients with congenital or acquired immunodeficiency and those with incomplete historical data were excluded.

Severe infection was considered to be indicated by the following critical conditions: (1) breathlessness and/or increased breathing rate (≥ 60 breaths/min); (2) neurological abnormalities: unresponsiveness, lethargy, agitation, convulsions etc; (3) persistent high fever for more than 3 days, accompanied by a severe cough and phlegm; (4) severe vomiting and diarrhea, with accompanying dehydration; (5) oliguria (a urinary output < 0.80 mL/(kg·h) or a daily urine volume < 200 mL/m^2^), or acute renal failure; (6) comorbid pneumonia; (7) without oxygen supplementation, the patient's pulse oxygen saturation (SpO2) < 92%; (8) clinical and hematological examination suggested the possibility of hemophagocytic syndrome; (9) the original underlying diseases were significantly aggravated; (10) other clinical conditions requiring hospitalization. A critical case refers to an influenza case accompanied by one of the following complications: (1) respiratory failure; (2) acute necrotizing encephalopathy (ANE); (3) shock; (4) multiple organ dysfunction; (5) other grave clinical conditions that necessitate ongoing monitoring and treatment.

A comprehensive data was collected, encompassing various demographic and clinical details such as sex, gestational week at birth, mode of delivery, birth weight, feeding patterns, age at admission, duration of hospitalization, season of onset, exposure to family members exhibited respiratory symptoms, maternal vaccination status during pregnancy, patient symptoms, physical signs, laboratory indicators, imaging findings, treatment methods, and outcomes. The diagnosis criteria for neonatal pneumonia rely on clinical presentation, radiological, or lung ultrasound findings.

The statistical analysis was conducted using SPSS 29.0. The Kolmogorov‐Smirnov nonparametric test was employed to ascertain whether the sample data followed a normal distribution, and *p* < 0.05 was considered to be statistically significant. Countable data were expressed as rates (%), whilst measurement data that conformed to a normal distribution were expressed as $\bar{\chi }\pm s$ and measurement data that did not conform to a normal distribution were expressed as M (Q1, Q3).

## 3 Results

A total of 26 neonates diagnosed with neonatal influenza by positive nasopharyngeal swab antigen tests and meeting the established inclusion criteria were enrolled in the study. All patients tested negative for SARS‐CoV‐2 in nasopharyngeal swab antigen tests. All neonates were delivered in the obstetrics department of our hospital. Each neonate was initially discharged home before later required hospitalization for influenza. Thirteen patients (50%) were delivered spontaneously, whereas 13 (50%) were delivered via cesarean section. The mean birth weight of the neonates was 3103.08 ± 402.52 g. The mean age at admission was 16.48 ± 7.93 days, including 4 (15.38%) neonates aged < 7 days, and 22 (84.62%) aged ≥ 7days, but ≤ 28 days. The patient population was predominantly male, with 15 patients (57.69%) being male and 11 patients (42.31%) being female. The study revealed that four patients (15.38%) were late preterm neonates, and 22 patients (84.62%) were term neonates. Fourteen patients (53.85%) were exclusively breastfed, whereas 12 patients (46.15%) were mixed feeding (Tables 1 and 2).

**Table 1:** Symptoms of neonatal influenza in preterm and term groups.

Groups	Fever	Nasal congestion	Neonatal jaundice	Cough	Sneezing	Sputum in the throat	Tachypnea	Rhinorrhea	Loss of appetite	Milk vomiting	Diarrhea	Abdominal bloting
Preterm (4)	3	2	2	1	0	0	0	0	1	2	0	0
Term (22)	11	10	10	9	3	2	2	1	7	1	1	1

*Note:* The number is cases of patients.

**Table 2:** Blood test results in preterm and term groups.

Groups	WBC > 15 × 10^9^/L	WBC < 5 × 10^9^/L	N% > 50%	L > 2 × 10^9^/L	L% > 50%	M% > 9.8%	CRP ≥ 10 mg/L	IL6 > 11 pg/mL	AST > 40 U/L	ALT > 40 U/L	CK > 200 U/L	CK‐MB > 24 U/L	LDH > 600 U/L	Lactic acid > 2 mmol/L
Preterm (4)	0	0	0	4	2	3	0	1	2	0	1	3	0	4
Term (22)	1	3	3	19	13	13	2	5	13	1	7	21	3	17

*Note:* The number is cases of patients, N% for neutrophil percentage, L for lymphocyte count, L% for lymphocyte percentage, M% for monocyte percentage.

**Figure 1:**
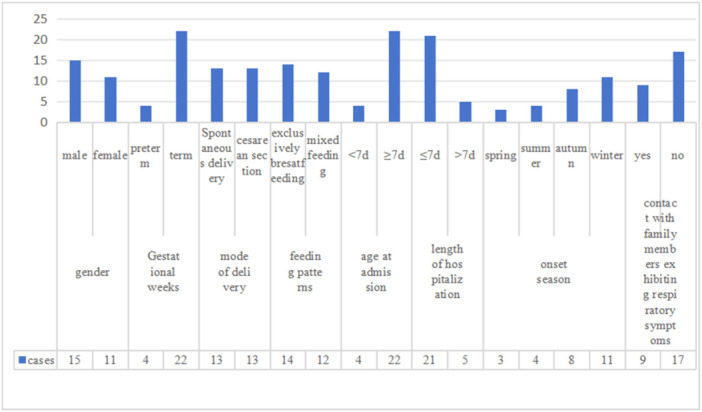
General data of neonates. *Note:* The number is cases of patients.

Twenty‐five patients were diagnosed with influenza A (96.15%), whereas one patient (3.85%) presented with a co‐infection of both influenza A and B. The duration of hospitalization ranged 3–12 days, in 21 neonates (80.77%) it was ≤ 7 days, and five (19.23%) it was > 7 days. The climate of Chengdu, Sichuan, is characterized by subtropical humid monsoon. Cases of illness were observed throughout the year, with 3 (11.54%) in spring, 4 (15.38%) in summer, 8 (30.77%) in autumn, and 11 (42.31%) in winter. Furthermore, nine patients (34.61%) had close contact with family members showing respiratory symptoms prior to hospitalization. None of the neonates' mothers received the influenza vaccine during the period of gestation (See Figure 1).

**Figure 2:**
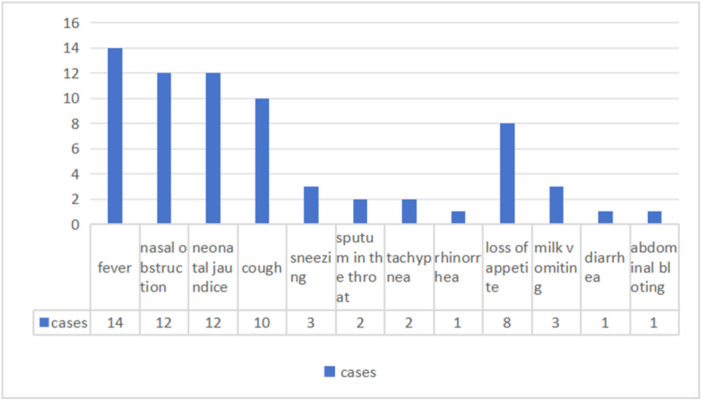
Symptoms of neonatal influenza. *Note:* The number is cases of patients, some patients had 2 or more symptoms at the same time.

Based on the grading criteria for influenza, two patients were identified as being in a severe condition due to tachypnea (≥ 60 breaths/min). All seven patients were successfully treated and discharged following their initially hospitalization for neonatal pneumonia, four of these cases were also complicated by preterm birth, but were later readmitted. The current study found that fever was the most common symptoms of neonatal influenza, with 14 neonates manifested fever. The peak temperatures of patients ranged from 37.5°C to 39.1°C. Specifically, 8 neonates exhibited temperatures ≤ 38.0°C, whereas 6 neonates exhibited temperatures > 38.1°C. The duration of fever exhibited a range from 0.5 to 3 days, with the mean duration being 1.16 ± 0.80 days. Other symptoms included nasal congestion, neonatal jaundice, cough, sneezing, sputum in the throat, tachypnea, rhinorrhea, loss of appetite, milk vomiting, diarrhea, and abdominal bloating (See Figure 2).

All patients underwent a comprehensive assessments, which included blood routine examinations, C‐reactive protein (CRP), biochemical tests, blood gas analysis, and blood culture. Additionally, certain patients underwent sputum culture or pathogen antibody testing. The results of the routine blood examinations revealed a total white blood cell count (WBC) ranged from 2.86 × 10^9^/L to 15.12 × 10^9^/L, decreased (< 5 × 10^9^/L) in 3 patients, and elevated > 15 × 10^9^/L in 1 patient. The neutrophil count ranged from 1.02 × 10^9^/L to 6.61 × 10^9^/L. The neutrophil percentage in neonatal influenza ranged from 11.6% to 73.0%, within 3 cases > 50%. The lymphocyte count ranged from 0.63 × 10^9^/L to 7.76 × 10^9^/L, within 23 cases > 2 × 10^9^/L. The lymphocyte percentage ranged from 20.3% to 75.8%, within 15 cases > 50%. The monocyte percentage ranged from 1% to 20.3%, within 16 cases > 9.8%. Two patients had elevated CRP ≥ 10 mg/L. Six patients had elevated interleukin‐6 (IL6) > 11 pg/mL. One patient had elevated procalcitonin (PCT) > 0.5 µg/L. The serum aspartate aminotransferase (AST) level of 15 neonates was found to be elevated (> 40 U/L), and the serum alanine aminotransferase (ALT) level of 1 neonate was elevated (> 40 U/L). Eight patients exhibited elevated creatine kinase (CK) level (> 200 U/L), furthermore, the creatine kinase isoenzyme MB (CK‐MB) level was elevated (> 25 U/L) in 24 patients. Lactate dehydrogenase (LDH) level was elevated (> 600 U/L) in 3 patients. Blood gas analysis revealed elevated level of lactic acid (> 2 mmol/L) in 21 patients.

Seventeen neonates diagnosed with influenza exhibited signs of neonatal pneumonia upon chest radiography or lung ultrasound examination. Six patients were co‐infected with other pathogens. Five of these patients had sputum cultures positive for *Klebsiella pneumoniae*, *Klebsiella oxytoca*, *Staphylococcus aureus*, or *Escherichia coli*. One neonate exhibited a positive antibody test for *Mycoplasma pneumoniae*. In contrast, blood cultures from all patients yielded negative results.

Fifteen patients underwent chest radiography, revealed increased reticular opacitics and interstitial markings in bilaterally lungs. Additionally, one patient exhibited slight thickening of the lobular fissure. Lung ultrasound was performed on four patients, with two showed increased B‐lines, other two were normal.

Four preterm neonates with influenza, whose corrected gestational age at admission ranged from 38 to 40 weeks, were treated with oseltamivir. The dosage of 1.5 mg/(kg·time) administered twice daily for 5 days. Twenty‐two term neonates with influenza were administered oseltamivir at a dosage of 3 mg/(kg·time) twice daily for 5 days. None of the patients reported any adverse drug reactions. None of the patients received intravenous peramivir. Among the patients, 14 were administered antibiotics; 13 received cefotaxime sodium, and 1 with amoxicillin clavulanate potassium. Subsequently, based on pathogen test results, one patient's antibiotic was changed to azithromycin. Two neonates underwent high‐flow nasal cannula (HFNC) oxygen therapy. Seventeen patients were administered cough medication along with atomization therapy. Additionally, twelve patients diagnosed with neonatal hyperbilirubinemia were treated with blue light therapy. Followed these treatments, all patients recovered. A telephone follow‐up, conducted 1 month post‐discharge, revealed a favorable prognosis for all patients.

## 4 Discussion

In the current study, 25 neonates were infected with influenza A virus, whereas one neonate was co‐infected with influenza A and B viruses. The study showed that influenza A is the most common type of influenza virus causing neonatal influenza, and the result is consistent with previous findings in neonates and children of other ages [5, 6, 7]. The majority (84.61%) of our patients were seen after more than 8 days of age, which supports other reports of the incubation period of influenza A ranging from 1–4 days [2]. In this study, influenza was observed to occur year‐round, with its highest incidence in winter and autumn. This pattern aligns with the characteristic epidemiology of influenza in subtropical humid monsoon climates [1, 2].

Neonatal influenza primarily results from horizontal transmission, as vertical transmission is rare. The most common route of infection involves close contact with symptomatic family members [2, 8]. The infection poses considerable risks to both pregnant women and infants, frequently resulting in severe disease, obstetric and adverse birth outcomes [9]. Maternal vaccination with the influenza vaccine not only reduces the risk of infection in pregnant women, but also transfers protective antibodies to the fetus against influenza virus infection [10]. Recent studies affirm that influenza vaccination during pregnancy is safe and shows no association with preterm birth, low birth weight, newborns small for gestational age, congenital abnormalities, spontaneous abortion, or stillbirth [11, 12, 13]. Despite this, influenza vaccine coverage among pregnant women in China is estimated to be below 1% [11, 14], substantially lower than the 71% rate in the United States [15], largely due to safety concerns and limited public awareness [11, 14]. Pregnant women face an elevated risk of severe illness and complications arising from influenza, especially in the second and third trimesters. Consequently, the administration of the influenza vaccine is advisable for pregnant women in any trimester of their pregnancy [1, 16, 17]. Enhancing training for healthcare providers and expanding public health education care are critical to improving vaccine uptake among pregnant women across all trimesters.

Fourteen neonates were exclusively breastfed while twelve were received mixed feeding. Breastfeeding strengthens neonatal immune defenses by supplying immunoglobulins and bioactive proteins, including secretory immunoglobulin A (SIgA), lactoferrin, Iysozyme, which reinforce intestinal mucosal integrity and support innate immunity. Maternal influenza vaccination additionally upregulates T‐cell marker genes such as CD44 and CD8A in breast milk, thereby enhancing adaptive immunity and lowering the incidence of infant respiratory infections [18, 19, 20, 21]. These findings underscore the critical importance of recommending breastfeeding.

In the study, we found that most neonates had normal peripheral WBC, CRP, and IL‐6 after influenza infection. Only one neonate showed an increased WBC, and three had decreased WBC. These observations are consistent with previous reports involving children of other age groups [22]. Following influenza virus infection, a decrease in lymphocytes is typically observed [23]. Nevertheless, our study revealed an unexpected outcome: 15 patients exhibited an elevated lymphocyte percentage > 50%, and 23 patients showed an increased lymphocyte count greater than 2 × 10^9^/L. This variation might be attributed to the distinct characteristics of white blood cell classification in neonates compared to children of other age groups. Sixteen (61.54%) patients had a monocyte percentage > 9.8%, which is consistent with previous reports. Monocyte are critical in antiviral immunity, and previous studies have shown a significant increase in monocyte among influenza patients compared to those with the common cold [23, 24]. An elevated monocyte percentage may suggest the presence of a viral infection, such as influenza. Additionally, some patients demonstrated elevated AST, ALT, CK, CK‐MB, and LDH levels.

The lobular changes observed in neonatal influenza‐induced pneumonia on chest radiography are similar to those observed in children of other age groups [25]. Lung ultrasound examination of two patients revealed an elevated lung B‐lines, indicating possible interstitial edema or inflammation. In diagnosing neonatal pneumonia, lung ultrasound demonstrates sensitivity and specificity comparable to chest radiography, yet avoids its associated radiation risks. This modality also differentiates accurately between viral and bacterial pneumonia. Therefore, lung ultrasound is useful in this study for the monitoring of neonates with pneumonia [26].

The present study revealed that fever was the most common symptom of neonatal influenza, aligns with the findings of prior reports [2]. The peak temperature ranged from 37.5°C to 39.1°C, and the duration of fever lasted between 0.5 and 3 days. This febrile period was shorter than that observed in other pediatric age groups, a finding consistent with a previous study [27]. This shorter duration likely stems from neonates' unique thermal characteristics, their multiple heat dissipation pathways, and their rapid rate of heat dissipation [28]. Two neonates presented with fever and loss of appetite but lacked respiratory symptoms, making it difficult to distinguish their condition from neonatal sepsis. Twelve neonates with influenza exhibited respiratory symptoms such as cough or nasal congestion, along with gastrointestinal symptoms including loss of appetite and milk vomiting; however, none presented with fever. The absence of distinct clinical signs in neonatal influenza complicates its differentiation from other respiratory infections, as its manifestations frequently mimic those of other atypical bacterial or viral infections, such as RSV, hMPV, SARS‐CoV‐2, parainfluenza virus, adenovirus, and Mycoplasma pneumoniae [29, 30, 31]. Six neonates were co‐infected with other pathogens, including *Klebsiella pneumoniae*, *Klebsiella oxytoca*, *Staphylococcus aureus*, *Mycoplasma pneumoniae*, and *Escherichia coli*. Two of these co‐infected neonates were in a critical condition and received high‐flow nasal cannula (HFNC) oxygen therapy. Consequently, the prompt detection of respiratory pathogens and the conduct of sputum cultures are imperative [32, 33].

Oseltamivir is the only neuraminidase inhibitor approved to treat influenza in both preterm and term neonates. Its most common adverse reactions are vomiting and epigastric discomfort [34]. The administration of oseltamivir for the treatment of influenza, initiated within 48 h of symptom onset, has been documented to reduce the course of the illness [1, 35, 36]. In our study, oseltamivir was given after 48 h in 11 patients. Overall, oseltamivir was well tolerated and our patients had favorable outcomes following therapy. Hence, its utilization is advisable even in such scenarios [2, 35, 36]. For pediatric patients who show a poor response to anti‐influenza therapy after 3 to 5 days or experience recurrent disease, accompanied by early signs of severe influenza, pediatricians must consider the possibility of concurrent bacterial infections and promptly administer antibiotics [37]. 14 patients underwent antibiotic treatment. Certain febrile neonates suspected of sepsis received antibiotics as part of their initial treatment course; however, these were promptly discontinued upon ruling out bacterial infection [38].

The prevention of influenza in neonates relies on proactive strategies, which include promoting the receipt of the influenza vaccine by family members, especially mothers during pregnancy [39, 40, 41]. Vaccinating healthcare workers is essential to reduce transmission to vulnerable groups like neonates and children, improve patient vaccine compliance, and strengthen pandemic preparedness. Therefore, it is important that NICU staff and family pediatricians are vaccinated against influenza [42].

Promoting breastfeeding, isolating influenza patients via droplet and contact precautions [43], and encouraging symptoms family members to adopt non‐pharmacological measures, including thorough comprehensive hand hygiene, mask‐wearing, and social distancing, effectively reduces viral transmission to neonates [1, 44, 45]. During influenza epidemic seasons, neonates with influenza‐like symptoms should receive timely testing to enable early case isolation and prevent nosocomial transmission [46, 47, 48]. Asymptomatic individuals have the potential to transmit the virus; therefore, testing should be considered for newborns exposed to infected family members or healthcare workers [49]. Pregnant women are recommended to receive influenza vaccination, which safeguards both maternal and neonatal health [1, 9, 10, 15]. Influenza vaccination is not recommended for newborns due to the immaturity of their immune systems and the specific immune response required for vaccine efficacy.

The study has several limitations, primarily due to its small sample size. Future research could expand upon these findings by conducting a more comprehensive comparative analysis of clinical data between neonates with influenza alone and those with co‐infections. Additionally, we plan to undertake follow‐up studies to determine whether influenza virus infection leaves any lasting impacts on this patient population.

## 5 Conclusions

In this single‐year study, influenza A was the predominant type identified, and is frequently observed in neonates ≥ 7 days of age. The clinical manifestations of neonatal influenza is non‐specific, fever 14/26 (53.85%) is the main symptom in the study. Most neonates with influenza exhibited normal WBC, CRP and IL‐6, whereas lymphocyte and monocyte percentages were commonly elevated. During influenza epidemics, timely laboratory tests and pathogen tests such as antigen test are needed for early recognition of neonatal influenza patients with fever, respiratory symptoms and/or gastrointestinal symptoms. Antiviral therapy with oseltamivir for neonatal influenza is safe, and demonstrating a favorable outcome. Breastfeeding is recommended. Strict infection control measures and prompt isolation constitute key strategies for preventing neonatal influenza virus infection.

## Author Contributions

M.Z., J.C., J.Z., Z.Y., J.F. and X.Q. collected and analyzed the medical data of neonatal influenza. M.Z. wrote the first draft of the manuscript and was the major contributor in writing the manuscript. M.Z. made critical revision of the article. All authors read and approved the final manuscript.

## Funding

The authors received no specific funding for this work.

## Ethics Statement

The study protocol received approval from the Ethics Committee of Chengdu Integrated TCM & Western Medicine Hospital.

## Consent

Written informed consent was obtained from the patient's guardian for publication.

## Conflicts of Interest

The authors declare no conflicts of interest.

## References

[1] T. M. Uyeki , D. S. Hui , M. Zambon , D. E. Wentworth , and A. S. Monto , “Influenza,” Lancet 400, no. 10353 (2022): 693–706, 10.1016/S0140-6736(22)00982-5. 36030813 PMC9411419

[2] X. Fu , J. Long , Y. Xiong , et al., “Epidemic Patterns of the Different Influenza Virus Types and Subtypes/Lineages for 10 Years in Chongqing, China, 2010‐2019,” Human Vaccines & Immunotherapeutics 20, no. 1 (2024): 2363076, 10.1080/21645515.2024.2363076. 38847280 PMC11164227

[3] R. M. Wolf and J. W. Antoon , “Influenza in Children and Adolescents: Epidemiology, Management, and Prevention,” Pediatrics in Review 44, no. 11 (2023): 605–617, 10.1542/pir.2023-005962. 37907421 PMC10676733

[4] R. Njouom , G. C. Monamele , B. Ermetal , et al., “Detection of Influenza C Virus Infection Among Hospitalized Patients, Cameroon,” Emerging Infectious Diseases 25, no. 3 (2019): 607–609, 10.3201/eid2503.181213. 30789339 PMC6390756

[5] M. M. Merișescu , M. Luminos , C. Pavelescu , and G. Jugulete , “Clinical Features and Outcomes of the Association of Co‐Infections in Children With Laboratory Confirmed Influenza During the 2022‐2023 Season: A Romanian Perspective,” Viruses 15, no. 10 (September 2023): 2035, 10.3390/v15102035. 37896811 PMC10611070

[6] C. Jia , W. Jia , X. Yi , et al., “Clinical Analysis of Influenza in the Neonatal Intensive Care Unit,” Italian Journal of Pediatrics 50, no. 1 (September 2024): 184, 10.1186/s13052-024-01742-6. 39294774 PMC11411831

[7] National Clinical Medical Research Center for Respiratory Diseases, Respiratory Group of the Chinese Medical Association Pediatrics , “Guidelines for the Diagnosis, Treatment and Prevention of Influenza in Children (2024 Physician Edition),” Chinese Journal of Practical Pediatrics 39, no. 12 (2024): 881–895, 10.3760/cma.j.cn101070-20241105-00719.

[8] V. Le Sage , A. C. Lowen , and S. S. Lakdawala , “Block the Spread: Barriers to Transmission of Influenza Viruses,” Annual Review of Virology 10, no. 1 (2023): 347–370, 10.1146/annurev-virology-111821-115447. 37308086

[9] C. Robinson , J. Oberye , J. van Boxmeer , et al., “A Prospective Cohort Study on Pregnancy Outcomes of Persons Immunized With a Seasonal Quadrivalent Inactivated Influenza Vaccine During Pregnancy,” Vaccines 10, no. 10 (September 2022): 1577, 10.3390/vaccines10101577. 36298442 PMC9611467

[10] M. K. Ellingson , M. Z. Dudley , R. J. Limaye , D. A. Salmon , S. T. O'Leary , and S. B. Omer , “Enhancing Uptake of Influenza Maternal Vaccine,” Expert Review of Vaccines 18, no. 2 (2019): 191–204, 10.1080/14760584.2019.1562907. 30587042 PMC6378696

[11] S. Zhou , C. M. Greene , Y. Song , et al., “Review of the Status and Challenges Associated With Increasing Influenza Vaccination Coverage Among Pregnant Women in China,” Human Vaccines & Immunotherapeutics 16, no. 3 (2020): 602–611. 31589548 10.1080/21645515.2019.1664230 PMC7227693

[12] H. Lee , D. Yoon , J. H. Kim , et al., “Association of Influenza Vaccination During Pregnancy With Health Outcomes in Mothers and Children: A Population‐Based Cohort Study,” Clinical Pharmacology & Therapeutics 117, no. 5 (2025): 1381–1392, 10.1002/cpt.3565. 39854110

[13] A. K. Regan and F. M. Munoz , “Efficacy and Safety of Influenza Vaccination During Pregnancy: Realizing the Potential of Maternal Influenza Immunization,” Expert Review of Vaccines 20, no. 6 (2021): 649–660, 10.1080/14760584.2021.1915138. 33832397

[14] Y. Song , T. Zhang , L. Chen , et al., “Increasing Seasonal Influenza Vaccination Among High Risk Groups in China: Do Community Healthcare Workers Have a Role to Play?,” Vaccine 35, no. 33 (2017): 4060–4063, 10.1016/j.vaccine.2017.06.054. 28668569 PMC8995106

[15] S. A. Irving , B. Crane , E. Weintraub , et al., “Influenza Vaccination Among Pregnant People Before and During the Coronavirus Disease 2019 (COVID‐19) Pandemic,” Obstetrics & Gynecology 142, no. 3 (2023): 636–639, 10.1097/AOG.0000000000005285. 37590982 PMC10868709

[16] L. C. Sahni , S. M. Olson , N. B. Halasa , et al., “Maternal Vaccine Effectiveness Against Influenza‐Associated Hospitalizations and Emergency Department Visits in Infants,” JAMA Pediatrics 178, no. 2 (2024): 176–184, 10.1001/jamapediatrics.2023.5639. 38109102 PMC10728798

[17] L. A. Grohskopf , J. M. Ferdinands , L. H. Blanton , K. R. Broder , and J. Loehr , “Prevention and Control of Seasonal Influenza With Vaccines: Recommendations of the Advisory Committee on Immunization Practices—United States, 2024‐25 Influenza Season,” MMWR: Recommendations and Reports 73, no. 5 (August 2024): 1–25, 10.15585/mmwr.rr7305a1. PMC11501009 39197095

[18] M. Zhang , H. Qiao , S. Yang , L. Y. Kwok , H. Zhang , and W. Zhang , “Human Breast Milk: The Role of Its Microbiota and Metabolites in Infant Health,” Journal of Agricultural and Food Chemistry 72, no. 19 (2024): 10665–10678, 10.1021/acs.jafc.3c07690. 38691667

[19] A. Laouar , “Maternal Leukocytes and Infant Immune Programming During Breastfeeding,” Trends in Immunology 41, no. 3 (2020): 225–239, 10.1016/j.it.2020.01.005. 32057705

[20] V. Demers‐Mathieu , R. K. Huston , A. M. Markell , E. A. McCulley , R. L. Martin , and D. C. Dallas , “Antenatal Influenza A‐Specific IgA, IgM, and IgG Antibodies in Mother's Own Breast Milk and Donor Breast Milk, and Gastric Contents and Stools From Preterm Infants,” Nutrients 11, no. 7 (July 2019): 1567, 10.3390/nu11071567. 31336756 PMC6682892

[21] V. Demers‐Mathieu , C. DaPra , and E. Medo , “Influenza Vaccine Associated With the Gene Expression of T Cell Surface Markers in Human Milk,” Breastfeeding Medicine 17, no. 3 (2022): 218–225, 10.1089/bfm.2021.0186. 34870443

[22] M. Villarreal , “Neutropenia: A Brief Review,” Pediatric Annals 52, no. 6 (2023): e238–e241, 10.3928/19382359-20230411-01. 37280003

[23] J. Chen , Y. Wang , M. Hong , et al., “Application of Peripheral Blood Routine Parameters in the Diagnosis of Influenza and *Mycoplasma pneumoniae* ,” Virology Journal 21, no. 1 (July 2024): 162, 10.1186/s12985-024-02429-4. 39044252 PMC11267962

[24] Y. Zheng and J. Zhu , “The Differential Significance of Elevated Monocytes in Influenza Patients,” Modern Practical Medicine 31, no. 9 (2019): 1176–1177, 10.3969/j.issn.1671-0800.2019.09.018.

[25] M. J. Choi , Y. S. Lee , J. Y. Lee , and K. S. Lee , “Novel Influenza A (H1N1) Virus Infection in Children: Chest Radiographic and CT Evaluation,” Korean Journal of Radiology 11, no. 6 (2010): 656–664, 10.3348/kjr.2010.11.6.656. 21076592 PMC2974228

[26] M. Beshara , E. A. Bittner , A. Goffi , L. Berra , and M. G. Chang , “Nuts and Bolts of Lung Ultrasound: Utility, Scanning Techniques, Protocols, and Findings in Common Pathologies,” Critical Care 28, no. 1 (October 2024): 328, 10.1186/s13054-024-05102-y. 39375782 PMC11460009

[27] J. Nayak , G. Hoy , and A. Gordon , “Influenza in Children,” Cold Spring Harbor Perspectives in Medicine 11, no. 1 (January 2021): a038430, 10.1101/cshperspect.a038430. 31871228 PMC7778215

[28] A. Abdul‐Mumin , N. A. A. Boi‐Dsane , S. T. Oladokun , S. A. Owusu , and P. Ansah , “A Retrospective Data Analysis on Prevalence and Risk Factors for Hypothermia Among Sick Neonates at Presentation to the Neonatal Intensive Care Unit of the Tamale Teaching Hospital,” PLoS One 19, no. 5 (May 2024): e0303159, 10.1371/journal.pone.0303159. 38753864 PMC11098396

[29] Z. Sun , L. Ke , Q. Zhao , et al., “The Use of Bioinformatics Methods to Identify the Effects of SARS‐CoV‐2 and Influenza Viruses on the Regulation of Gene Expression in Patients,” Frontiers in Immunology 14 (February 2023): 1098688, 10.3389/fimmu.2023.1098688. 36911695 PMC9992716

[30] B. A. Sullivan and R. W. Grundmeier , “Machine Learning Models as Early Warning Systems for Neonatal Infection,” Clinics in Perinatology 52, no. 1 (2025): 167–183, 10.1016/j.clp.2024.10.011. 39892951 PMC13150861

[31] R. Falsaperla , V. Sortino , D. La Cognata , et al., “Acute Respiratory Tract Infections (ARTIs) in Children After COVID‐19‐Related Social Distancing: An Epidemiological Study in a Single Center of Southern Italy,” Diagnostics 14, no. 13 (June 2024): 1341, 10.3390/diagnostics14131341. 39001232 PMC11240751

[32] E. Roth , M. Cattaneo , Y. Hollenstein , et al., “The Impact of Rapid Molecular Diagnostics for Influenza on Antibiotic Stewardship in the Emergency Department‐An Observational Retrospective Study,” Antibiotics (USSR) 14, no. 2 (January 2025): 120, 10.3390/antibiotics14020120. PMC11851583 40001364

[33] T. W. Clark , K. Lindsley , T. B. Wigmosta , et al., “Rapid Multiplex PCR for Respiratory Viruses Reduces Time to Result and Improves Clinical Care: Results of a Systematic Review and Meta‐Analysis,” Journal of Infection 86, no. 5 (2023): 462–475, 10.1016/j.jinf.2023.03.005. 36906153

[34] H. R. Kang , E. K. Lee , W. J. Kim , and J. Y. Shin , “Risk of Neuropsychiatric Adverse Events Associated With the Use of Oseltamivir: A Nationwide Population‐Based Case‐Crossover Study,” Journal of Antimicrobial Chemotherapy 74, no. 2 (2019): 453–461, 10.1093/jac/dky445. 30418537

[35] R. E. Malosh , E. T. Martin , T. Heikkinen , W. A. Brooks , R. J. Whitley , and A. S. Monto , “Efficacy and Safety of Oseltamivir in Children: Systematic Review and Individual Patient Data Meta‐Analysis of Randomized Controlled Trials,” Clinical Infectious Diseases 66, no. 10 (2018): 1492–1500, 10.1093/cid/cix1040. 29186364

[36] A. M. Frutos , H. M. Ahmad , D. Ujamaa , et al., “Underutilization of Influenza Antiviral Treatment Among Children and Adolescents at Higher Risk for Influenza‐Associated Complications—United States, 2023‐2024,” MMWR. Morbidity and Mortality Weekly Report 73, no. 45 (November 2024): 1022–1029, 10.15585/mmwr.mm7345a2. 39541236 PMC11576051

[37] M. Qiao , G. Moyes , F. Zhu , Y. Li , and X. Wang , “The Prevalence of Influenza Bacterial Co‐Infection and Its Role in Disease Severity: A Systematic Review and Meta‐Analysis,” Journal of Global Health 13 (June 2023): 04063, 10.7189/jogh.13.04063. 37319008 PMC10270314

[38] J. Gyllensvärd , M. Studahl , L. Gustavsson , et al., “Antibiotic Use in Late Preterm and Full‐Term Newborns,” JAMA Network Open 7, no. 3 (March 2024): e243362, 10.1001/jamanetworkopen.2024.3362. 38517437 PMC10960197

[39] I. Kristinsdottir , A. Haraldsson , and V. Thors , “Influenza Vaccination in Pregnant Women in Iceland 2010–2020 and the Burden of Influenza in Pregnant Women and Their Infants,” Vaccine 42, no. 8 (2024): 2051–2058, 10.1016/j.vaccine.2024.02.046. 38413277

[40] E. A. Clemens , B. C. Holbrook , B. McNeilly , M. Kanekiyo , B. S. Graham , and M. A. Alexander‐Miller , “TLR Agonists Induce Sustained IgG to Hemagglutinin Stem and Modulate T Cells Following Newborn Vaccination,” NPJ Vaccines 7, no. 1 (August 2022): 102, 10.1038/s41541-022-00523-8. 36038596 PMC9424286

[41] H. Shaikh , P. Koli , V. Undale , et al., “Safety and Protective Effects of Influenza Vaccination in Pregnant Women on Pregnancy and Birth Outcomes in Pune, India: A Cross‐Sectional Study,” Vaccines 11, no. 6 (May 2023): 1034, 10.3390/vaccines11061034. 37376423 PMC10305502

[42] D. Coulibaly , A. Douba , K. N'guessan , et al., “Intent to Receive Flu Vaccine and Influenza Vaccination Coverage Among Health Professionals During 2019, 2020 and 2021 Campaigns in Côte D'ivoire,” supplement, Vaccine 42 no. S4 (2024): 126076, 10.1016/j.vaccine.2024.06.043. 38926071 PMC11464181

[43] P. S. Pannaraj , A. G. da Costa‐Martins , C. Cerini , et al., “Molecular Alterations in Human Milk in Simulated Maternal Nasal Mucosal Infection With Live Attenuated Influenza Vaccination,” Mucosal Immunology 15, no. 5 (2022): 1040–1047, 10.1038/s41385-022-00537-4. 35739193 PMC9225800

[44] A. Holz , “Ideological Consistency and News Sharing as Predictors of Masking Among College Students,” International Journal of Environmental Research and Public Health 21, no. 12 (December 2024): 1652, 10.3390/ijerph21121652. 39767491 PMC11675830

[45] T. Jefferson , L. Dooley , E. Ferroni , et al., “Physical Interventions to Interrupt or Reduce the Spread of Respiratory Viruses,” Cochrane Database of Systematic Reviews 1, no. 1 (January 2023): 006207, 10.1002/14651858.CD006207.pub6. 20091588

[46] M. Hanna , R. Shah , L. Marquez , R. Barzegar , A. Gordon , and M. Pammi , “Infant Isolation and Cohorting for Preventing or Reducing Transmission of Healthcare‐Associated Infections in Neonatal Units,” Cochrane Database of Systematic Reviews 6, no. 6 (June 2023): 012458, 10.1002/14651858.CD012458.pub2. PMC10297826 37368649

[47] J. W. Antoon , J. Z. Amarin , O. Hamdan , et al., “Antiviral Use Among Children Hospitalized With Laboratory‐Confirmed Influenza Illness: A Prospective, Multicenter Surveillance Study,” Clinical Infectious Diseases (December 2024): ciae573, 10.1093/cid/ciae573. PMC12497963 39688383

[48] M. P. Montgomery , S. E. Morris , M. A. Rolfes , et al., “The Role of Asymptomatic Infections in Influenza Transmission: What Do We Really Know,” Lancet Infectious Diseases 24, no. 6 (2024): e394–e404, 10.1016/S1473-3099(23)00619-9. 38128563 PMC11127787

[49] T. A. Becerra‐Culqui , D. Getahun , V. Chiu , L. S. Sy , and H. F. Tseng , “Prenatal Influenza Vaccination or Influenza Infection and Autism Spectrum Disorder in Offspring,” Clinical Infectious Diseases 75, no. 7 (2022): 1140–1148, 10.1093/cid/ciac101. 35174388

